# α-Pinene, a Main Component of *Pinus* Essential Oils, Enhances the Expression of Insulin-Sensitive Glucose Transporter Type 4 in Murine Skeletal Muscle Cells

**DOI:** 10.3390/ijms25021252

**Published:** 2024-01-19

**Authors:** Giordana Feriotto, Federico Tagliati, Valentina Costa, Marcello Monesi, Claudio Tabolacci, Simone Beninati, Carlo Mischiati

**Affiliations:** 1 Department of Chemical, Pharmaceutical and Agricultural Sciences, University of Ferrara, 44121 Ferrara, Italy; giordana.feriotto@unife.it; 2 Department of Neurosciences and Rehabilitation, University of Ferrara, 44121 Ferrara, Italy; federico.tagliati@unife.it; 3 Department of Environmental Sciences and Prevention, University of Ferrara, 44121 Ferrara, Italy; valentina.costa@unife.it; 4 UOC (Unità Operativa Complessa) Territorial Diabetology, AUSL Ferrara, 44121 Ferrara, Italy; m.monesi@ospfe.it; 5 Research Coordination and Support Service, Superior Institute of Health, 00161 Rome, Italy; claudio.tabolacci@iss.it; 6 Department of Biology, University of Rome “Tor Vergata”, 00133 Rome, Italy; beninati@bio.uniroma2.it

**Keywords:** glucose transporter-4, essential oils, α-pinene

## Abstract

Glucose transporter-4 (GLUT4) represents the major glucose transporter isoform responsible for glucose uptake into insulin-sensitive cells, primarily in skeletal muscle and adipose tissues. In insulin-resistant conditions, such as type 2 diabetes mellitus, GLUT4 expression and/or translocation to the cell plasma membrane is reduced, compromising cell energy metabolism. Therefore, the use of synthetic or naturally occurring molecules able to stimulate GLUT4 expression represents a good tool for alternative treatments of insulin resistance. The present study aimed to investigate the effects of essential oils (EOs) derived from *Pinus* spp. (*P. nigra* and *P. radiata*) and of their main terpenoid constituents (α- and β-pinene) on the expression/translocation of GLUT4 in myoblast C2C12 murine cells. For this purpose, the chemical profiles of the EOs were first analyzed through gas chromatography–mass spectrometry (GC-MS). Cell viability was assessed by MTT assay, and GLUT4 expression/translocation was evaluated through RT-qPCR and flow cytometry analyses. The results showed that only the *P. nigra* essential oil (PnEO) and α-pinene can increase the transcription of the *Glut4/Scl2a4* gene, resulting in a subsequent increase in the amount of GLUT4 produced and its plasma membrane localization. Moreover, the PnEO or α-pinene can induce *Glut4* expression both during myogenesis and in myotubes. In summary, the PnEO and α-pinene emulate insulin’s effect on the GLUT4 transporter expression and its translocation to the muscle cell surface.

## 1. Introduction

Type 2 diabetes mellitus (T2DM) is the most widespread type of diabetes and the fourth leading cause of death in developed countries. Approximately 5% of the world population suffers from this chronic metabolic disorder, marked by a progressive worsening of carbohydrate and lipid metabolism profiles, as well as a state of hyperglycemia. This is largely due to peripheral insulin resistance, increased hepatic glucose production, and deficient insulin production [1].

The GLUT family of glucose transporters are integral membrane proteins that possess 12 transmembrane helices which emerge on both the cytoplasmic and outer sides of the plasma membrane. The various isoforms of this transporter are expressed differently in various tissues and play different roles in glucose metabolism due to their varying substrate specificities, transport kinetics, and expression, which can be regulated depending on physiological conditions [2]. To date, 14 members of the GLUT/SLC2 (solute carrier family 2) have been identified [3,4]. The SCL2 member 4 (*Scl2a4*) gene, more commonly known as *Glut4*, encodes the glucose transporter GLUT4, which is stimulated by insulin in skeletal muscle, cardiac, and adipose tissues. When insulin levels are low, GLUT4 is stored in intracellular GLUT4 storage vesicles (GSVs) or, to a lesser extent, stored in other cellular compartments as a reserve. When insulin is released into circulation from pancreatic β cells, it binds to the insulin receptor and triggers the redistribution of GLUT4 from GSVs to the cell surface plasma membrane to maintain glycemic homeostasis [5]. In insulin-resistant conditions, such as diabetes, the trafficking of GLUT4-containing vesicles is impaired [6], and the increase in GLUT4 expression and/or its translocation to the cell surface is a primary target in the treatment of diabetes [7,8].

It has been demonstrated that a range of medicinal plants and fruit- and vegetable-based foods can intervene at critical stages of glucose metabolism and could potentially be used to develop new anti-diabetic medications [9,10]. There are multiple mechanisms underlying their anti-diabetic effects, including intervening with the insulin receptor signaling pathways, the translocation of GLUT4 receptors, the activation of PPAR-γ, or the reduction of intestinal glucose absorption [11]. For centuries, essential oils (EOs) have been employed for medicinal and traditional practices. Terpenes and terpenoids, which constitute the EO, are a small group of molecules that possess physicochemical properties which enable them to penetrate the skin and reach the subcutaneous tissue and bloodstream, and they are considered safe and effective. As a result, they are utilized to enhance the skin penetration of drugs [12]. In addition, EOs have been proposed as an additional treatment option for diabetes [13,14] and its associated complications [15]. EOs possess antioxidant properties and can protect against oxidative stress by neutralizing free radicals, regulating antioxidant enzymes, and reducing lipid peroxidation, due to their varied chemical composition [15]. Furthermore, EOs have potential hypoglycemic benefits attributed to their capacity to increase glucose uptake, reduce glucose production, and enhance insulin sensitivity in diabetes [13].

In this work, we sought to analyze the effects of EOs derived from plants of the genus *Pinus* and of their main terpenoids on the expression and translocation of GLUT4 in skeletal muscle C2C12 cells.

## 2. Results

### 2.1. Analysis of the Effect of Pinus EOs on Glut4 Gene Transcription in Myoblasts

**Figure 1:**
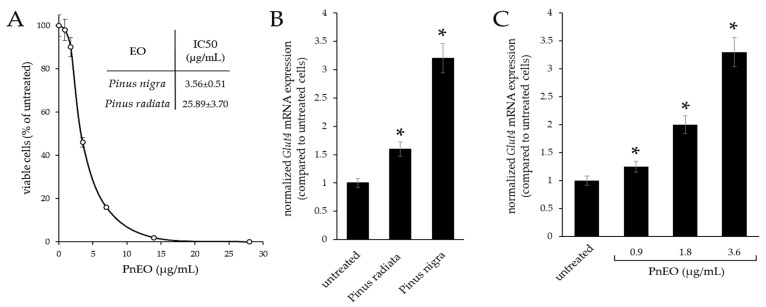
Effects of the *Pinus* spp. essential oils on *Glut4* mRNA expression. (**A**) C2C12 cells were exposed to increasing doses of the *P. radiata* (PrEO) or *P. nigra* (PnEO) essential oils for 72 h; cell viability was determined by MTT assay. (**B**) RT-qPCR analysis of *Glut4* mRNA expression in C2C12 cells treated with the PrEO and PnEO. (**C**) The PnEO regulated the mRNA expression of *Glut4* in C2C12 cells in a dose-dependent manner. Asterisks indicate significant values with respect to control untreated cells (*p* < 0.05).

During a preliminary phase, the cytotoxicity of the EOs from *Pinus radiata* (PrEO) and *P. nigra* (PnEO) was evaluated in C2C12 cells by MTT assay, and the IC_50_ values are reported in Figure 1A. Then, to test the biological activity of the EOs after administering them directly into a culture medium for 3 days at the IC_50_ concentration, *Glut4* expression was evaluated by RT-qPCR (Figure 1B). The PnEO showed the greatest increase compared to untreated cells, with a 3.2-fold increase, whereas the PrEO had a much lower effect, only resulting in a 1.5-fold increase. The study of the effects of the PnEO on *Glut4* mRNA expression was further refined, and the results suggest that the PnEO can increase expression in a dose-dependent manner (Figure 1C).

### 2.2. Effect of the PnEO on Cell Surface Exposure of the GLUT4 Transporter

**Figure 2:**
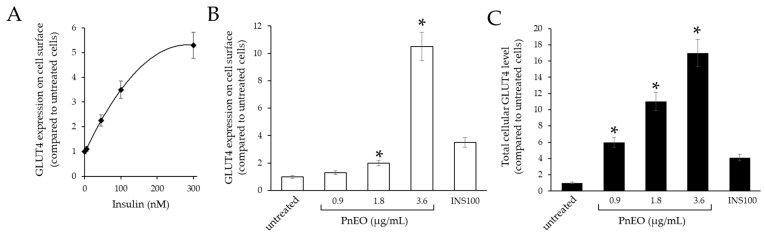
Treatment with the PnEO increases the number of GLUT4 transporters present on the C2C12 cell surface. Data are reported as median fluorescence values of the populations. (**A**) Dose-dependent effect of human insulin on the exposure of GLUT4 on the cell surface. (**B**) Dose-dependent effect of the PnEO on the exposure of GLUT4 on the cell surface. INS100, 100 nM insulin. (**C**) Dose-dependent effect of the PnEO on total GLUT4 expression (cell surface + GSV). INS100, 100 nM insulin. Asterisks indicate significant values relative to control untreated cells (*p* < 0.05).

A preliminary analysis, conducted using flow cytometry and an α-GLUT4 antibody, showed that C2C12 cells respond to exposure to human insulin for 30 min in a dose-dependent manner, leading to GLUT4 expression on the cell surface (Figure 2A). These data are consistent with observations outlined in the literature, which have demonstrated that in C2C12 cells, a significant rise in glucose absorption occurs after exposure to 100 nM insulin for 30 min [16]. GLUT4 levels increased linearly from 0 to 100 nM insulin, rising to around 3.5 times their original value. However, once the insulin concentration went above 300 nM, a plateau effect was noted. Afterward, 100 nM insulin was administered as a positive control to assess GLUT4 expression. Following this, the effect of the PnEO on a dose-dependent basis was studied at doses both cytotoxic (3.6 µg/mL) and lower than cytotoxic (0.9 and 1.8 µg/mL). To define the amount of GLUT4 exposed on the cell surface, intact cells were immune-decorated and analyzed (Figure 2B), while the total amount of GLUT4 present in the cellular compartments was determined by permeabilizing the cells before immune decoration (Figure 2C).

Analysis of the data in Figure 2B revealed that when the PnEO was administered at the IC50 concentration, the exposure of GLUT4 on the cell surface markedly increased to more than 10 times that of untreated cells; however, lesser effects were observed at lower concentrations. Data from Figure 2C on permeabilized cells demonstrated an increase in total GLUT4 protein correlating with dose, suggesting that the PnEO may influence GLUT4 neo-synthesis similarly to insulin. This inference was in accordance with the increased *Glut4* gene transcription observed in Figure 1C.

### 2.3. Characterization of the Chemical Composition of the PnEO

GC-MS analysis was performed on the PnEO and PrEO to determine their constituent terpenes. The list is reported in Table 1.

The two EOs have a few similar terpenes, and they differ in the amounts of each. α- and β-pinene are the most abundant terpenes in the PnEO and PrEO, respectively. They are the main and most common molecules in both EOs, together representing 65–74% of the total terpenes present. It is widely accepted that, despite the complexity of the EOs, which are composed of more than 20 components, their biological activity could be due to the few terpenes present in high concentrations [17]. Therefore, the influence of the PnEO and PrEO on GLUT4 expression may be attributable to one or both of these terpenes. GC-MS analysis revealed that the percentage of α- and β-pinene was overturned in the two EOs, 65.3% and 8.3%, respectively, in the PnEO and 23.4% and 42.6% in the PrEO. The differing percentage distributions of these terpenes could be the explanation for the different induction activities of the two EOs on glucose transporter expression. Therefore, we focused subsequent experiments on the effects of both terpenes on biological activity.

### 2.4. Effect of Pinenes on GLUT4 Expression

**Figure 3:**
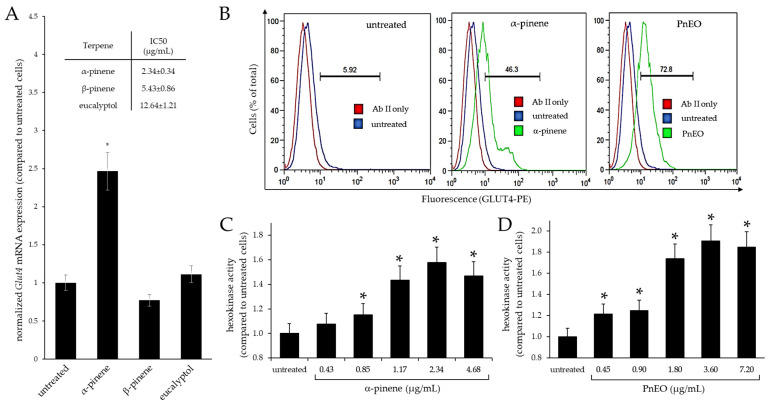
Effects of principal terpenes found in the *Pinus* essential oils on GLUT4 expression. (**A**) RT-qPCR analysis of *Glut4* mRNA expression in C2C12 cells treated with α-pinene, β-pinene, and eucalyptol. (**B**) A representative flow cytometric analysis of GLUT4 protein in C2C12 cells treated with α-pinene and the PnEO or in untreated cells. (**C**,**D**) Hexokinase I activity in C2C12 cells treated with α-pinene in (**C**) and the PnEO in (**D**). Asterisks indicate significant values relative to control untreated cells (*p* < 0.05).

The cytotoxicity of α-pinene, β-pinene, and eucalyptol (a terpene absent in the two EOs) was evaluated in C2C12 cells, and a preliminary experiment was performed at IC50 to study their influences on *Glut4* gene expression by RT-qPCR analysis (Figure 3A). Only α-pinene increased *Glut4* transcripts by approximately 2.5 times (*p* < 0.05) compared to untreated cells, while β-pinene and eucalyptol did not cause any significant inductions. These data indicate that α-pinene is an active ingredient of the PnEO causing the observed effect on *Glut4* gene expression.

Using flow cytometry, the expression of the GLUT4 protein was evaluated on the cell surface of proliferating C2C12 cells treated with α-pinene and compared to the expression produced by the treatment with the PnEO as control, both administered at the IC50 concentration (Figure 3B). While in untreated cells the presence of GLUT4 on the cellular surface was low (median value = 5.92), both treatments with α-pinene and the PnEO increased it (median values, respectively, of 46.3 and 72.8). The inability of α-pinene to reach GLUT4 induction values comparable to those obtained with the PnEO suggests that, even if this terpene is certainly the main ingredient responsible for the effect, the EO’s other terpenes collaborate to determine the final score. Further studies are necessary to identify the other terpenes necessary to obtain maximal GLUT4 exposure.

In addition, the effect of treatment with either α-pinene (Figure 3C) or the PnEO (Figure 3D) on hexokinase activity was evaluated as an indicator of increased glucose uptake into the cell. Skeletal muscle expresses two isoforms of hexokinase (type I and type II), but in the absence of insulin stimulation, hexokinase I and II make up 70–75% and 25–30% of the total hexokinase activity, respectively [18]. Therefore, the activity of hexokinase I was assessed in skeletal muscle C2C12 cells. The results indicate that both α-pinene and the PnEO led to a dose-dependent increase in enzyme activity, with increases of 60% and 80%, respectively, in comparison to the untreated control cells. This indicates that the PnEO or α-pinene help C2C12 cells to absorb and process glucose. The effect of the PnEO being stronger than that of α-pinene further suggests that minor terpenes play an additional part.

### 2.5. Effects of the PnEO and α-Pinene on Glut4 Expression in Myotubes

**Figure 4:**
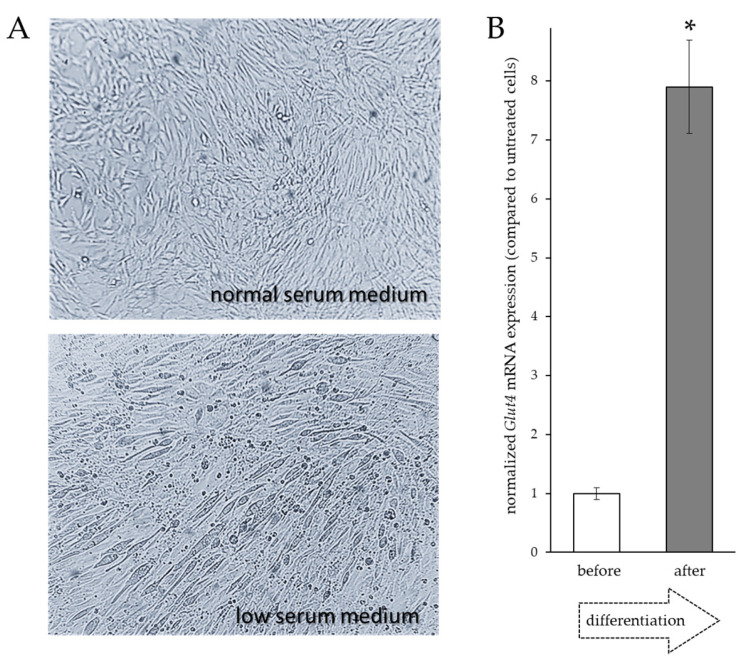
Expression of *Glut4* in differentiated C2C12 cells. (**A**) Representative light microscopy images of three experiments reproduced in triplicate of undifferentiated (day zero) and differentiated (day 14) C2C12 cells. (**B**) RT-qPCR analysis of *Glut4* mRNA expression in C2C12-differentiated cells. The asterisk indicates the significant value obtained after differentiation compared to untreated control cells (*p* < 0.05).

Myotubes were obtained from C2C12 cells cultured for 14 days in low serum differentiating medium. As shown in Figure 4A, after 14 days in this medium, the morphological analysis showed signs indicative of muscle differentiation, such as the formation of elongated and multinucleated cellular syncytia (myotubes).

In the RT-qPCR analysis shown in Figure 4B, the level of *Glut4* transcripts increased approximately 8-fold in myotubes compared to undifferentiated cells.

**Figure 5:**
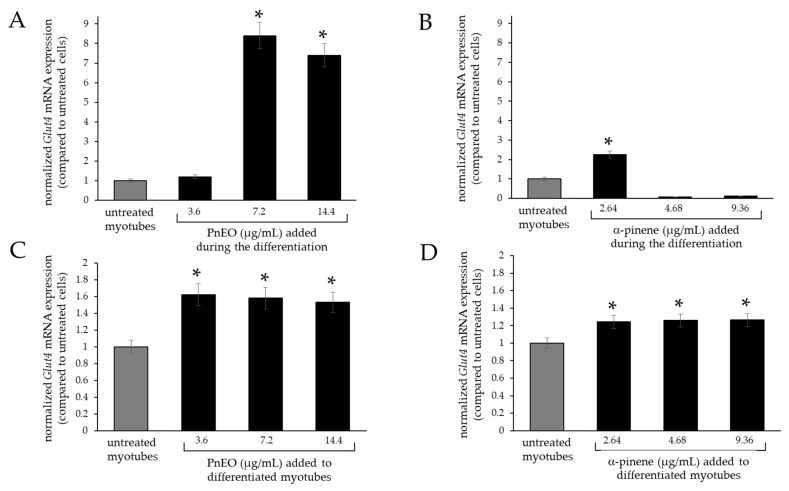
RT-qPCR analysis of *Glut4* mRNA expression in C2C12 cells in different experimental settings. (**A**,**B**) Effects of the PnEO in (**A**) and α-pinene in (**B**) addition at day zero of differentiation. (**C**,**D**) Effects of the PnEO (**C**) and α-pinene (**D**) addition after C2C12 myogenic differentiation. The level of transcripts, normalized to the housekeeping gene, refers to control untreated cells (grey column). Asterisks indicate significant values relative to control untreated cells (*p* < 0.05).

Therefore, the effects of the PnEO and α-pinene on *Glut4* transcripts were evaluated by RT-qPCR in two different experimental settings, by adding them to the cells before or after differentiation (Figure 5).

Upon the addition of the PnEO to the differentiating medium on day zero of myogenesis, there was no morphological difference in the myotubes from those obtained without the EO. However, there was an approximately 8.5-fold increase in *Glut4* gene expression in the myotubes treated with the PnEO when compared to those not treated (Figure 5A). Similarly, cells differentiated normally in the presence of α-pinene, but this terpene had a lesser effect on *Glut4* expression compared to the PnEO, increasing up to a maximum of 2.2-fold (Figure 5B). In differentiated myotubes, when the PnEO was added for an additional three days post-myogenesis, a smaller increase in expression was attained, reaching a maximum of 1.6-fold compared to untreated myotubes (Figure 5C). In the same condition, α-pinene yielded even less, with a maximum of 1.2-fold (Figure 5D).

## 3. Discussion

Our findings indicate that the PnEO and α-pinene can affect glucose transport within muscle cells through dual actions. Firstly, as seen in Figure 1B,C and Figure 3A, they increase the transcription of the *Glut4* gene, leading to an increase in the total amount of GLUT4 protein, as evidenced in tests with the PnEO (Figure 2C). Secondly, the PnEO and α-pinene promote the relocation of the GLUT4 glucose transporter protein to the cell membrane (Figure 2B and Figure 3B), resulting in higher glucose consumption than in control untreated cells (Figure 3C,D). Ultimately, treatments with the PnEO or α-pinene reproduce the outcomes of the insulin interaction with its receptor (Figure 2). Additionally, neither the PnEO nor α-pinene affect the regular progression of myogenesis. Thus, these substances should not hinder the usual recovery functions of skeletal muscle fibers caused by muscle damage and/or physical exercise. Similarly to the effects produced in myoblasts, the expression of *Glut4* transcripts increased when either PnEO or α-pinene was administered during myogenesis (Figure 5A,B) or after, directly to myotubes (Figure 5C,D), as compared to that observed in their absence (Figure 4B). The rise in *Glut4* expression suggests a potential boost in the absorption of glucose by muscle cells, which can help regulate the blood sugar levels in the body. In summary, the PnEO and α-pinene emulate insulin’s effect on GLUT4 transporter expression and translocation from the GSVs to the muscle fiber surface, thereby increasing the uptake of glucose.

Even if the specific mechanism behind the biological effect of the PnEO and α-pinene that we have observed remains elusive, literature has demonstrated that α-pinene can interact with lipids of the cell membrane without inducing significant cellular changes [19]. We propose that this interaction could facilitate the fusion of GSVs into the cytoplasmic membrane. With regard to a possible mechanism of action put in place by the PnEO and its main terpene to enhance *Glut4* gene transcription, we have no data. However, it has been known that insulin enhances *Glut4* gene expression in the skeletal muscle by activating AT-rich and E-box elements in a PI3K/AKT-dependent mechanism, and by repressing NF-κB-site activity [8]. Furthermore, it has been demonstrated that α-pinene increases IκB-α (a regulatory protein that inhibits the activity of NF-κB) protein levels in THP-1 monocytes, thus inhibiting NF-κB activity [20]. Therefore, as a tentative hypothesis, we suggest that α-pinene could act by inhibiting NF-κB activity in C2C12 cells, in turn enhancing *Glut4* gene transcription. These topics will be the subject of our future investigations.

As described above, α-pinene is a permeant vehicle useful for transdermal drug delivery [12]. This terpene interacts with lipids in the outermost layer of skin, known as the stratum corneum, to increase the movement of hydrophilic molecules through the skin barrier without resulting in significant changes in the cute upon treatment [19]. Furthermore, in this work, we demonstrated the potential of α-pinene to regulate glucose levels. Based on these characteristics, the future possible use of α-pinene as a vehicle for administering anti-diabetic drugs may result in a more powerful and efficient strategy for managing diabetes. In addition, the employment of α-pinene can increase the production of antioxidant enzymes at the cellular level, enhancing overall antioxidant capabilities to better counteract the harmful effects of oxygen-free radicals on lipid peroxidation [21]. Ultimately, this means that α-pinene has the potential to mitigate the complications associated with elevated oxidative stress found in diabetic tissues [22].

## 4. Materials and Methods

### 4.1. Materials

Commercial essential oils from *Pinus radiata* (genus *Pinus*, subgenus *Pinus*, section *Trifoliae*, subgenus *Attenuata*) (PrEO) and *Pinus nigra* (genus *Pinus*, subgenus *Pinus*, section *Pinus*, subsection *Pinus*) (PnEO) were obtained from a local grocery. α-pinene, β-pinene, and eucalyptol were obtained from FLUKA, Milan, Italy. Stock solutions were stored in the dark at −80 °C in a glass vial sealed with a Teflon cap until their use. Insulin degludec Tresiba^®^ was purchased from Novo Nordisk A/S (Bagsværd, Denmark) and stored at +4 °C in the dark.

### 4.2. Cell Cultures

C2C12 is an immortalized mouse myoblast cell line, a subclone of myoblasts originally obtained by Yaffe and Saxel [23]. The cells were maintained as myoblasts in a humidified atmosphere of 5% CO_2_ air in Dulbecco’s modified Eagle’s medium (DMEM) and 10% fetal bovine serum, penicillin (50 units/mL) and streptomycin (50 mg/mL). Myotubes from C2C12 cells were obtained as described by Murakami and coworkers [24]. Briefly, when the cells reached confluence, the medium was replaced with a medium containing DMEM and 2% horse serum, thus marking day zero of differentiation. Then, the medium was replaced every 3–4 days. Usually, by the fourteenth day, the cells had fused into skeletal muscle myotubes.

### 4.3. Cell Viability

C2C12 cells were treated in the presence or absence of scalar concentrations of the tested compound for 72 h. Then, cell viability was measured by MTT assay as previously described [25]. Half-maximal inhibitory concentration (IC50) was calculated using GraphPad Prism version 6.01 (GraphPad Software, San Diego, CA, USA). At least three independent experiments were performed in triplicate.

### 4.4. RNA Extraction

Total RNA was extracted using TRizol Reagent following the manufacturer’s instructions (Thermo Fisher, Milan, Italy) as previously described [25]. Briefly, cells were lysed in 0.5 mL of TRIzol reagent, and 200 μL of chloroform was added. After centrifugation, the aqueous phase was collected, and the RNA was precipitated by isopropanol addition. The RNA pellet was rinsed in 75% ethanol and rehydrated in TE (10 mM Tris, 1 mM EDTA, pH8) at 55 °C for 10 min.

### 4.5. Quantitative Real-Time Polymerase Chain Reaction (RT-qPCR)

Reverse transcription (RT) quantitative PCR (qPCR) assay was performed as previously specified [25]. To assess mouse *Glut4* gene expression, the following primers were used: forward 5′-GCC ATC TTG ATG ACC GTG GCT C-3′ and reverse 5′-GGC AGC TGA GAT CTG GTC AAA CG-3′. Mouse *Hprt* (Hypoxanthine-guanine phosphoribosyltransferase) expression, considered as the endogenous control, was analyzed using the following primers: forward 5′-GTC CCA GCG TCG TGA TTA GCG-3′ and reverse 5′- AGA GGT CCT TTT TCA CCA GCA AGC-3′. A 96-well optical plate containing reaction mixture was heated for 1.5 min at 95 °C, followed by 40 cycles of PCR, consisting of 30 s at 95 °C, 30 s at 68 °C and 30 s at 72 °C using a StepOnePlus™ Real-Time PCR System (Thermofisher, Milan, Italy). Reactions were performed in a 20 μL volume containing 10 μL SYBR green iTaq™ Universal SYBR^®^ Green Supermix (BioRad, Milan, Italy) containing the ROX internal passive reference dye, 0.5 μM of each primer, and optimized MgCl_2_ concentration between 1.5 and 3 mM. Endpoint amplified products were subjected to melt curve analyses to confirm that a single product was amplified in each well. The comparative ΔCT method was used for relative quantitation of gene expression. At least two independent experiments were performed in triplicates. Data analysis was performed by using StepOnePlus™ Software 2.3 (Thermofisher, Milan, Italy).

### 4.6. GC–MS Analysis

The GC–MS analysis was performed as described previously [26]. Briefly, the EOs were analyzed using an Agilent 5973 Network quadrupole mass selective detector (Agilent Technologies, Palo Alto, CA, USA) coupled with an Agilent GC 6850 Series II Network Trace gas chromatograph. An HP-5MS capillary column containing 5%-phenyl-methylpolysiloxane (30 m × 0.25 mm, film thickness, 0.25 μm) was employed. GC operating conditions were as follows: carrier gas, helium with a flow rate of 2 mL/min; column temperature program ranged from 45 °C to 100 °C at 1 °C/min, and then from 100 °C to 250 °C at 5 °C/min; injector inlet temperature, 280 °C; volume injected, 1 μL of the EO in dichloromethane; split ratio, 1:40. MS-operating parameters were as follows: ionization potential, 70 eV; ion source temperature, 230 °C; quadrupole temperature, 150 °C; solvent delay, 4.20 min; mass range, 35–300 *m*/*z*. The GC retention and mass spectra of peaks obtained were compared with those of authentic standards from the NIST/EPA/NIH database and mass spectra from the literature.

### 4.7. Hexokinase Activity

C2C12 cells were lysed for 20 min on ice in a solution containing 0.1% Triton X100 in PBS, and centrifuged for 5 min at 4500× *g*. The nuclei were discarded while the supernatant was processed. Samples containing 0.8–2.5 mg protein were added to a final volume of 100 µL of assay solution (20 mM Tris-HCl pH 7.5, 20 mM MgCl_2_, 4 mM EDTA, one unit/mL G6PDH, 10 mM glucose, 0.6 mM NADP+ and 5 mM ATP). Hexokinase (HK) activity was measured at 30 °C every minute for 30 min at 340 nm by spectrophotometry. All samples were assayed in triplicate, and each experiment was performed at least two times. Sample protein concentration was determined by spectrophotometry using Bradford’s reagent.

### 4.8. Flow Cytometry

The cells were treated with or without the tested compound for 72 h. Then, they were collected for the following analysis. Briefly, formalin-fixed cells were blocked at room temperature for 30 min in 1% bovine serum albumin (BSA) solution in PBS. Whole cells and cells permeabilized by cold absolute methanol for 15 min were stained with antibodies against GLUT4 (Origene, Rockville, MD, USA) (1:100) and then with phycoerythrin-conjugated anti-rabbit secondary antibodies (1:200) (Thermofisher, Milan, Italy). The samples were analyzed using FACS Canto II (Becton, Dickinson, ND, USA). Data analysis was performed using FlowJo software, version 9.9.6 (Tree Star, Inc., Ashland, OR, USA). The experiments were carried out in triplicate for each data point.

### 4.9. Statistical Analysis

The results were expressed as the arithmetic mean ± standard deviation. Statistical calculations were performed using a one-way ANOVA, and the differences among groups were examined using the Bonferroni *t*-test using GraphPad Prism. *p* value < 0.05 was considered significant.

## 5. Conclusions

This research is the first to report on the inducing effect of α-pinene on GLUT4 expression in the existing literature. However, based on our findings, it is evident that α-pinene is not able to completely reproduce the biological activity of the PnEO. This suggests that other minority terpenes, in combination with α-pinene, may play a crucial role in determining the overall impact of the EO. Hence, more investigations will be required to identify which specific minority terpenes have the potential to amplify the effect of α-pinene.

## Figures and Tables

**Table 1:** GC-MS analysis of the constituent terpenes in the *Pinus* spp. EOs. The two essential oils have similar terpenes (underlined), the most abundant of which are highlighted in bold.

*Pinus radiata*				*Pinus nigra*			
Peak	Compound	Area (%)	SD (%)	Peak	Compound	Area (%)	SD (%)
**1**	α-phellandrene	0.126	0.005	**1**	santene	0.164	0.006
**2**	α-pinene	23.416	0.890	**2**	tricyclene	0.622	0.024
**3**	camphene	0.790	0.030	**3**	**α-pinene**	**65.308**	2.482
**4**	**β-pinene**	**42.635**	1.620	**4**	camphene	5.981	0.227
**5**	β-myrcene	1.900	0.072	**5**	β-pinene	8.270	0.314
**6**	3-thujene	0.158	0.006	**6**	limonene	4.185	0.159
**7**	3-carene	8.319	0.316	**7**	α-pinene epoxide	0.949	0.036
**8**	limonene	11.663	0.443	**8**	cis-verbenol	3.223	0.122
**9**	β-trans-ocimene	0.925	0.035	**9**	α-campholenal	0.425	0.016
**10**	τ-terpinen	0.526	0.020	**10**	pinocarveol	0.935	0.036
**11**	terpinolen	2.133	0.081	**11**	cis-verbenol	2.558	0.097
**12**	linalool	0.358	0.014	**12**	α-terpineol	0.883	0.034
**13**	fenchol	0.254	0.010	**13**	myrtenol	0.421	0.016
**14**	pinocarveol	0.333	0.013	**14**	l-verbenone	0.728	0.028
**15**	L-isopulegol	0.218	0.008	**15**	cis-carveol	0.502	0.019
**16**	1-terpinen-4-ol	1.526	0.058	**16**	(-)-bornyl acetate	3.863	0.147
**17**	α-terpineol	2.893	0.110	**17**	τ-cadinene	0.638	0.024
**18**	(R)-(+)-β-citronellol	0.988	0.038	**18**	caryophyllene oxide	0.352	0.013
**19**	caryophyllene	0.191	0.007				
**20**	cadinene	0.465	0.018				
**21**	spathulenol	0.182	0.007				

## Data Availability

Data are contained within the article.
